# Epstein-Barr Virus (EBV) Encoded Dutpase Exacerbates the Immune pathology of Lupus Nephritis *In Vivo*

**DOI:** 10.23937/2378-3672/1410023

**Published:** 2016-08-20

**Authors:** Nicholas A Young, Marshall V Williams, Wael N Jarjour, Michael S Bruss, Brad Bolon, Samir Parikh, Anjali Satoskar, Maria Eugenia Ariza

**Affiliations:** 1 Division of Rheumatology and Immunology, Wexner Medical Center, The Ohio State University, USA; 2 Department of Internal Medicine, Wexner Medical Center, The Ohio State University, US; 3 Department of Molecular Virology, Immunology and Medical Genetics, The Ohio State University, USA; 4 Institute for Behavioral Medicine Research, The Ohio State University, USA; 5 Comparative Pathology and Mouse Phenotyping Shared Resource, Comprehensive Cancer Center, The Ohio State University, USA; 6 Department of Nephrology, Wexner Medical Center, The Ohio State University, USA; 7 Department of Pathology, Wexner Medical center, The Ohio State University, USA

**Keywords:** Lupus nephritis, Epstein-Barr virus, Deoxyuridine triphosphate nucleotidohydrolase, Interleukin-17, Autoimmunity

## Abstract

Epstein-Barr virus (EBV) is an environmental factor with strong links to systemic lupus erythematous (SLE) pathogenesis; however, the mechanism(s) remains unclear. The goal of this study was to determine whether the EBV protein “deoxyuridine triphosphate nucleotidohydrolase (dUTPase)”, which can induce aberrant immune responses, contributes to the immunopathology of lupus nephritis (LN). Using the NZM2410/J SLE mouse model, we demonstrated that intramuscular injections of EBV-dUTPase protein (10 μg) significantly enhanced glomerulonephritis compared to PBS-injected controls. The inflammation was characterized by interstitial/tubular cellular infiltrates, as well as increased IgG complex formation and C3 deposition in glomeruli. Additional immunohistochemical analyses revealed that EBV-dUTPase strongly induced IL-17 in glomeruli and tubules. More importantly, examination of kidney biopsies from class III/IV LN patients demonstrated the presence of EBV-dUTPase in infiltrating plasma-cell aggregates near glomeruli, where neighboring cells with increased TLR-2 and IL-17 expression were observed, suggesting EBV-dUTPase may exacerbate the immunopathologies in some LN patients.

## Introduction

Systemic lupus erythematous (SLE) is a complex autoimmune disease characterized by loss of self-tolerance to an array of self-antigens, primarily nuclear proteins, resulting in autoimmunity [1]. Lupus nephritis (LN) is the most common solid organ manifestation observed in SLE patients and poor renal function is a predictor of overall survival. The biochemical and cellular processes leading to LN involve alterations in clearance of dead cells, activation of antiviral immunity pathways and aberrant lymphocyte proliferation. This spectrum of changes results in immune complex (IC) deposition in glomeruli and tubulointerstitium, infiltration of inflammatory cells into the kidneys and subsequent production of cytokines and chemokines, which in turn trigger the onset and/or progression of renal pathology [1–3]. If untreated, the prognosis for LN is poor and clinical complications include irreversible organ failure [2,4].

Numerous serological studies have attempted to establish a causal relationship between Epstein-Barr virus (EBV) infection and the development of SLE [5–9] or SLE/LN [10]. Other studies demonstrated that antibodies directed against Epstein-Barr nuclear antigen 1 (EBNA-1) cross react with autoantigens associated with SLE [11] as well as dsDNA [12]. Furthermore, an increased EBV viral load in SLE patients was also demonstrated [13–15]. Whether or not the increased viral titers and EBV-specific immune responses observed in SLE reflect the sensitivity of the virus to perturbations in the immune system in these patients [16], and thus, represent a consequence of SLE rather than its cause, remains a topic of controversy. However, what is important to consider is that dysregulation of immune responses against EBV, specially, in individuals genetically predisposed to/or with the autoimmune disease SLE/LN, can trigger auto- reactive immunopathologies in these patients. Therefore, the potential role(s) of EBV as driver/promoter of the pathophysiological changes observed in some SLE LN patients with over- reactive immune systems remains unclear and warrants further investigation.

We have previously shown that the deoxyuridine triphosphate nucleotidohydrolase (dUTPase) encoded by EBV possesses novel immunomodulatory functions, independent of its conventional enzymatic activity, in part by triggering Toll-like receptor 2 (TLR2) mediated activation of NF-κB signaling in human embryonic kidney cells and immune cells [17–21]. These studies demonstrated that the EBV-dUTPase: (i) induce the secretion of the pro-inflammatory T_H_1/T_H_17 cytokines IL-6, IL-1β and type-I IFN in a TLR2-dependent manner [17,18,21], (ii) up-regulates the expression of the inflammatory BIC/microRNA-155 [18], and (iii) alters EBV-specific CD8+ T-cell function by up-regulating the expression of several genes involved with the antiviral immune response [18]. These findings have also been reported in patients with SLE [13–15,22]. The data presented in this study demonstrate that the EBV- dUTPase may play a role in the immune over-reactivity observed in a subset of patients with LN by triggering abnormal innate and adaptive immune responses thus, inducing an immune amplification cascade that promotes autoimmunity.

## Materials and Methods

### Purification of EBV-dUTPase

The recombinant EBV-dUTPase was purified using HisPurTM spin columns (Thermo Scientific; Rockford, IL), as we have described [23]. The purified recombinant protein was tested for the presence of contaminants as described [19] and was free of detectable levels of LPS, peptidoglycan, DNA and RNA.

### Animals

Female NZM2410/J mice were purchased from The Jackson Laboratories (Bar Harbor ME). All mice were housed at The Ohio State University Wexner Medical Center (OSUWMC) in a Biosafety Level 3 barrier facility on a 12-hour light/dark cycle, with chow and water available *ad libitum*. The facilities were maintained at 22-23°C and 30-50% relative humidity. All experiments were performed according to federal and institutional ethical guidelines on animal care and the animal protocol for this study was approved by the Institutional Animal Care and Use Committee at OSUWMC.

### Treatment regimens

Female (16-weeks old) NZM2410/J mice were weighed and blood from the submandibular vein was collected to obtain baseline measures for total body-weight and blood urea nitrogen (BUN) concentrations, respectively. Animals (n = 10 mice per treatment group; experiments run in duplicate) were then injected with purified recombinant EBV-dUTPase protein (10 μg) or PBS control intramuscularly (i.m; 0.2 mL) in the hind limb bi-weekly and evaluated daily for signs of clinical disease. Animals’ body-weight and BUN concentration were measured weekly. BUN (a marker for kidney function) levels were determined in serum using BUN Enzymatic Kit (Bioo Scientific Corporation, Austin, TX) according to the manufacturer’s protocol. Mice with BUN concentrations greater than 50 mg/dL and/or weight-loss over 20% relative to baseline measurements were euthanized and the kidneys collected for histological analysis.

### Mouse renal histopathology

Isolated kidney slices were fixed in 10% neutral buffered formalin, and processed into paraffin. Sections (4 μm) were stained with hematoxylin (Richard Allen Scientific Hematoxylin; Thermo Scientific, Waltham, MA) and eosin (Eosin-Y; Thermo Scientific) (H&E) using a Leica Autostainer (Leica Biosystems, Buffalo Grove, IL) for evaluation of general tissue architecture as previously described [24]. Inflammation severity was scored blinded by a board-certified veterinary pathologist (BB) using the 10x objective of a bright-field light microscope. Scores where: 0 = within normal limits, 1 = minimal change, 2 = mild change, 3 = moderate change and 4 = marked change. Digital images of 10 random fields/areas were obtained by photomicroscopy (Leica Microsystems; original objective magnifications: 20 × and 40 ×).

### Human renal tissue

Kidney biopsy tissue was obtained from six SLE patients with LN who were classified by a board-certified medical pathologist (AS) as class III or IV and who were undergoing LN flares. All study subjects provided written informed consent through an approved protocol for human subjects by the Institutional Review Board at OSUWMC.

### Immunohistochemistry

Immunohistochemistry was performed at OSU Comparative Pathology and Mouse Phenotyping Shared Resource and the OSUWMC Pathology Core Facilities. Immunoperoxidase staining of kidney sections was performed on formalin–fixed, paraffin-embedded sections (4 *μ*m) from EBV-dUTPase treated or PBS control mice. Briefly, tissue sections were de-paraffinized, rehydrated and then subjected to antigen retrieval by heat-induced epitope retrieval (HIER) (Dako S1699; pH 6.0; Carpinteria, CA) for 25 minutes at 96°C using a vegetable steamer (Black & Decker) and cooled for 15 minutes. Slides were stained with the following polyclonal primary antibodies: rat anti-mouse F4/80 (1:200; AbD Serotec, Raleigh, NC), rat anti-mouse C3 (1:50; Abcam, San Francisco, CA), goat anti-mouse IgG (1:60,000; Jackson Immuno research, West Grove, PA), rabbit anti-mouse TLR2 (1:500; Abcam, San Francisco, CA), or rabbit anti-mouse IL-17 (1:300; Abcam, San Francisco, CA) for 1 h at room temperature (RT) using the Intellipath Autostainer Immunostaining instrument. Rat, goat, or rabbit pre-immune serum were used as negative controls.

Similarly, formalin-fixed, paraffin-embedded kidney tissue sections from patients with class III or IV LN were stained with the following primary antibodies: mouse anti-human plasma cell (1/300; Abcam), rabbit anti-EBV-dUTPase (1/400; NeoBioLab, Cambridge, MA), rabbit anti-human TLR2 (1:400), or rabbit anti-human IL17 (1:300, Abcam, San Francisco, CA) specific primary Abs. The immunohistochemistry protocol employed for human tissues was equivalent to that described above for mouse tissues. Mouse isotype control antibody or rabbit pre-immune serum were used as negative controls. For both mouse and human kidneys, the stained tissue sections were washed and incu-bated with appropriate secondary antibodies for 30 min at RT. Procedures conducted with rabbit and mouse primaries used MACH 2TM horseradish peroxidase (HRP)-conjugated secondary antibodies (Biocare Medical, Concord, CA). Immunostains performed with rat and goat primaries utilized rabbit anti-goat (Abcam, San Francisco, CA) in 2% normal goat serum (Vector Labs, Burlingame, CA), and goat anti-rat (Abcam, San Francisco, CA) HRP-conjugated secondary antibodies. Bound antibodies were visualized by incubation with 3,3-diaminobenzidine chromogen (liquid DAB+; Dako, Carpinteria, CA) for 5’ at RT. Kidney slides were then counterstained with Richard Allen hematoxylin, dehydrated, cleared and mounted. All slides were scanned using the Aperio ScanScope XT eSlide capture device (Aperio, Vista CA) as described [25]. Quantitative analysis of inflammation was performed using the Aperio Digital Image Analysis software (v9.1) and exported to Microsoft Excel (v2010). Results are expressed as the mean pixel intensity of positive staining.

### Statistics

Statistical analysis was performed using a two-tailed Mann-Whitney non-parametric U-test using Prism version 6 statistical software. Results were considered statistically significant when *p* ≤ 0.05.

## Results

### EBV-dUTPase significantly enhanced the severity of glomerulonephritis in a mouse model of lupus nephritis

EBV is an environmental factor with strong links to SLE pathogenesis; however, a mechanism by which EBV may contribute to SLE and/or LN remains unclear. We have previously demonstrated that the dUTPase, which is expressed during lytic and abortive-lytic replication of EBV, possesses novel functions, independent of its enzymatic activity, in innate and adaptive immunity and can induce aberrant inflammatory responses, a hallmark of SLE. To investigate the role of EBV-dUTPase in the pathogenesis of LN, NZM2410/J mice were injected with EBV-dUTPase bi-weekly beginning at 16 weeks of age. Kidneys were collected from mice with BUN levels > 50 mg/dL and/or weight-loss exceeding 20% for H&E staining and histopathological scoring, as described in Materials & Methods.

**Figure 1:**
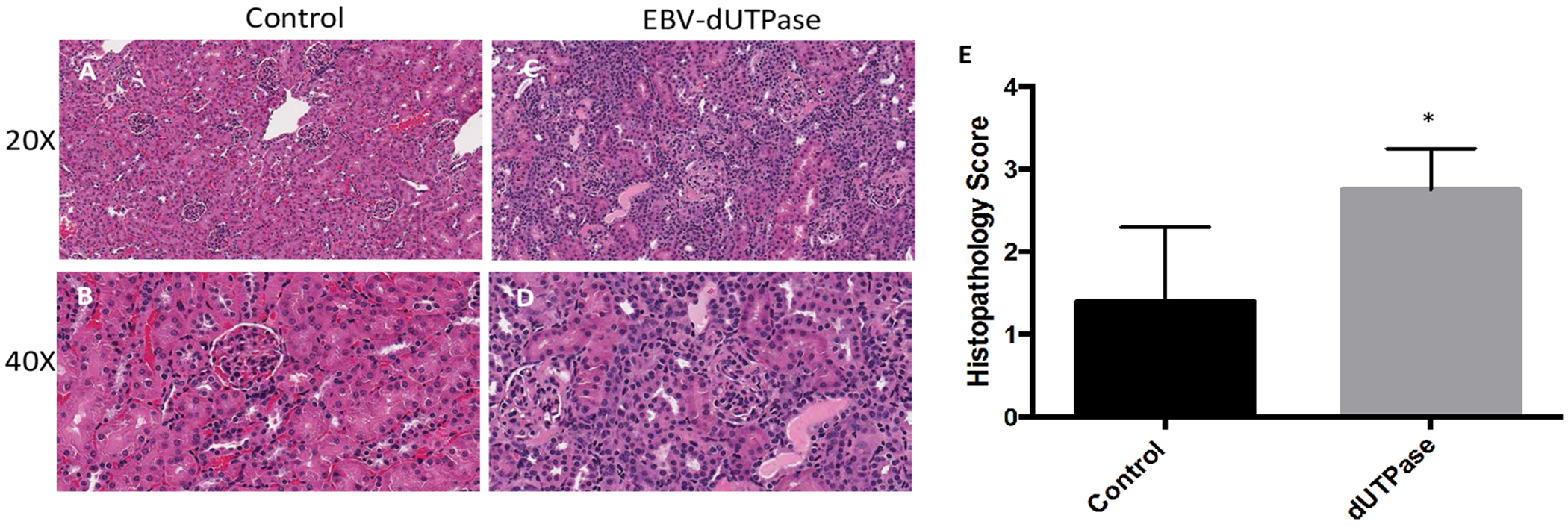
EBV-dUTPase enhances glomerulonephritis *in vivo*. Hematoxylin and Eosin (H&E) staining of formalin-fixed paraffin-embedded kidney sections from PBS-injected control (A,B) EBV-dUTPase-treated (C,D) NZM2410/J mice. Original objective magnification: 20 × (A,C); 40 × (B,D). (E) Semi-quantitative analysis of coded (“blinded”) histopathology scores for renal inflammation. Values represent the mean histopathology score ± SD of n = 10 mice per treatment group; *p = 0.0317 versus PBS control.

As shown in figure 1, mice injected with EBV-dUTPase had significantly enhanced renal inflammation characterized by robust induction of tubulointerstitial nephritis, abnormal glomerular morphology and hypercellularity, as well as a reduced Bowman’s capsule compared to PBS-injected controls (panels A-Panels D). Blinded analysis of histopathology scores for renal damage revealed significantly higher scores in EBV-dUTPase injected mice (mean ± SD score of 2.75 ± 0.5 on a 4-point scale) than in control mice (1.4 ± 0.5) (Figure 1E, *p* = 0.0317).

### EBV-dUTPase-mediated induction of renal pathology correlates with increased macrophage infiltration

**Figure 2:**
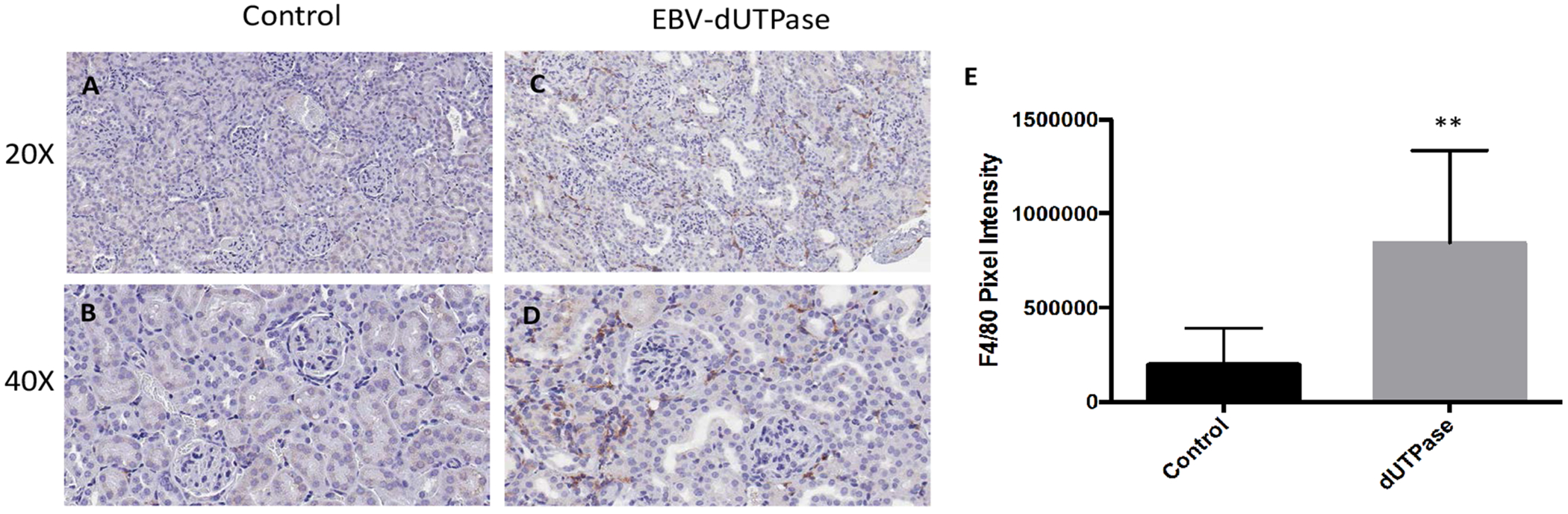
EBV-dUTPase induces macrophage infiltration into the inflamed kidneys of NZM2410/J mice. Immunohistochemical staining of formalin-fixed paraffin-embedded kidney sections from control (A,B) and EBV-dUTPase-injected (C,D) mice using an antibody to the macrophage surface marker F4/80. Hematoxylin counterstain. Original objective magnification: 20 × (A,C); 40 × (B,D). (E) Semi-quantitative analysis of F4/80 expression was performed using Aperio digital image analysis software. Values represent the mean pixel intensity of positive staining ± SD of n = 10 mice per treatment group; **p = 0.0012 versus PBS control.

To determine if EBV-dUTPase could contribute to the inflammatory pathology of LN by inducing macrophage infiltration, immunohistochemical staining of kidney sections was performed using an antibody to the macrophage cell marker F4/80. As shown in figure 2, immunohistochemical analysis revealed a statistically significant increase in macrophage infiltration in renal specimens from EBV-dUTPase injected mice relative to the controls (panels A-D, *p* = 0.0012). Quantitation of macrophage infiltration was determined using the Aperio Digital Image Analysis software (v9.1), as we have previously described [25] and demonstrated a pronounced increase in expression of F4/80 in renal tissue (Figure 2E).

### EBV-dUTPase increases IgG and complex complement component C3 deposition in the kidneys of NZM2410/J mice

**Figure 3:**
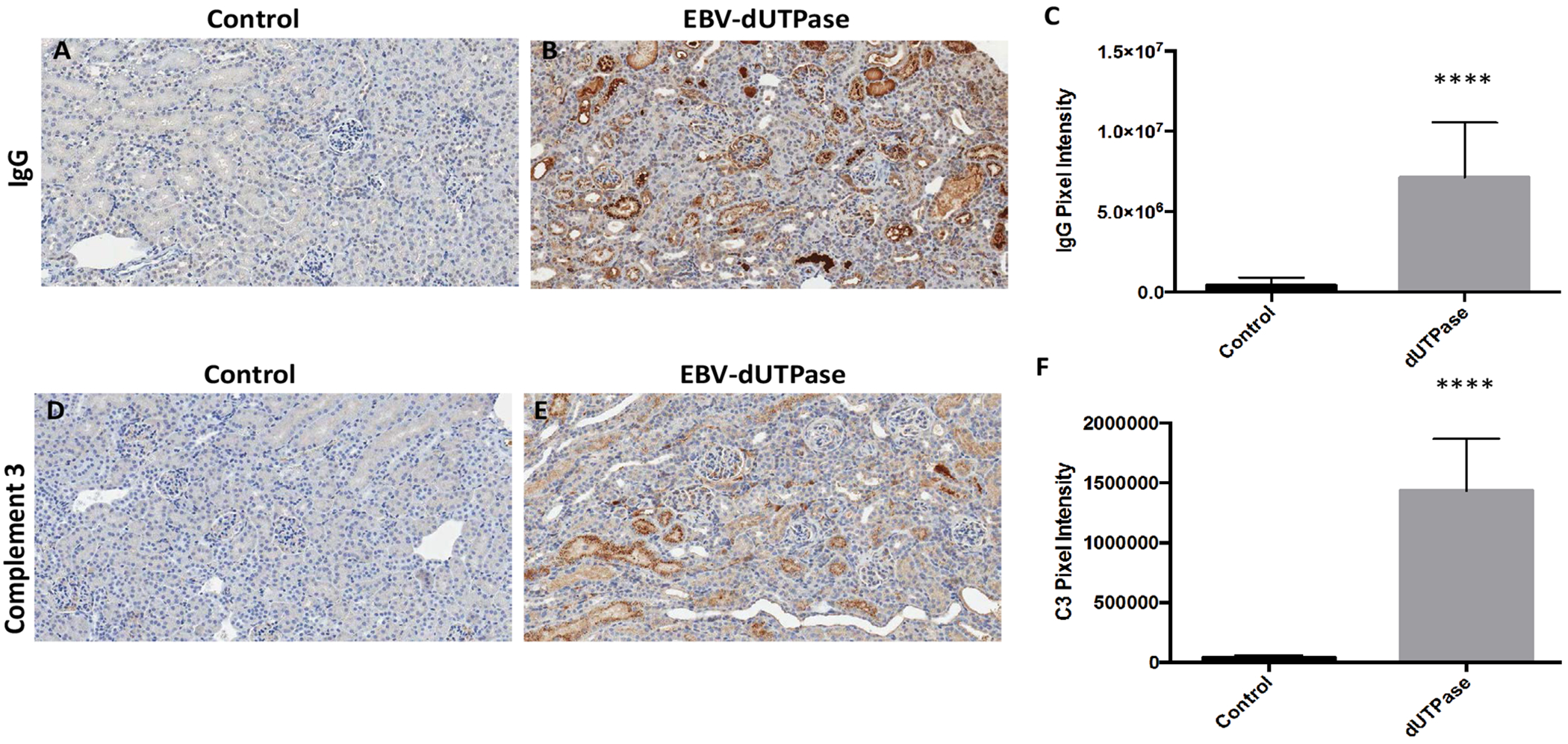
EBV-dUTPase protein strongly induces IgG complex formation and C3 fragment deposition in the inflamed kidneys of NZM2410/J mice. Immunohistochemical staining of kidney sections from control and EBV-dUTPase-injected mice using goat-α-IgG antibody (A,B) or rat-α-C3 antibody (D,E). Hematoxylin counterstain. Original objective magnification: 20×. Semi-quantitative analysis of kidney sections using Aperio digital image analysis demonstrated significant differences in the level of IgG complex formation (C) and C3 deposition (F) betwe treatment groups. Values represent the mean pixel intensity of positive staining ± SD of n = 10 mice per treatment group; ****p < 0.0001 versus saline control.

We next investigated whether or not the EBV-dUTPase mediated renal damage is associated with immune complex deposition, immunohistochemical staining for IgG was performed on kidney sections from EBV-dUTPase or PBS injected NZM2410/J mice. The severity and extent of IgG glomerular and tubular deposition in the kidney were markedly greater in EBV-dUTPase injected mice compared to PBS-injected mice (Figure 3A and Figure 3B). Quantitative analysis of IgG demonstrated significantly greater deposition in EBV-dUTPase injected mice (*p* < 0.0001; Figure 3C).

Activation of the complement inflammatory cascade leading to deposition of C3 fragments on target cells is associated with renal injury in LN. To determine whether the EBV-dUTPase protein contributes to the pathogenesis of LN by inducing C3 complex deposition in renal tissue, we evaluated kidney sections by immunohistochemistry to demonstrate localization of C3. As shown in figure 3, renal sections of EBV-dUTPase treated mice had significantly higher C3 deposition levels in glomeruli and tubules (Figure 3E) compared to controls (Figure 3D), (*p* < 0.0001; Figure 3F).

### EBV-dUTPase up-regulates TLR2 in NZM2410/J treated mice

**Figure 4:**
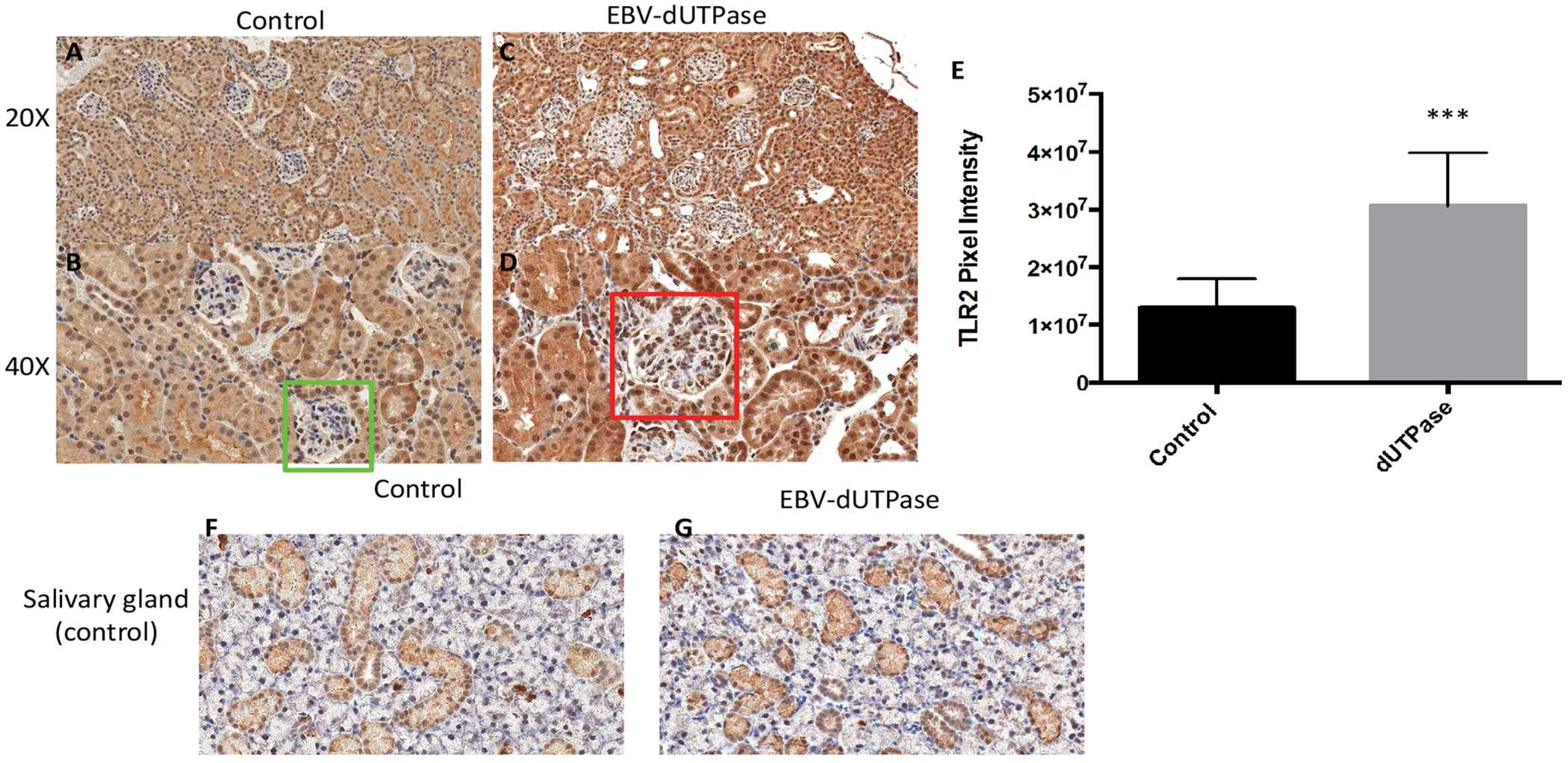
EBV-dUTPase up-regulates TLR2 protein expression in the glomerulus of NZM2410/J mice. Kidney sections from PBS control (A,B) and EBV-dUTPase-injected (C,D) mice were stained with a polyclonal rabbit-α-TLR2 antibody. Hematoxylin counterstain. Original objective magnification: 20 × (A,C); 40 × (B,D). (E) Semi-quantitative analysis of kidney sections using Aperio digital image analysis demonstrated significant differences in the level of TLR2 protein expression in the glomeruli between EBV-dUTPase-treated (red box) and PBS-injected mice (green box). Values represent the mean pixel intensity of positive staining ± SD of n = 5 mice per treatment group; ***p = 0.0001 versus PBS-injected control. Tubules in the salivary glands of PBS control (F) and EBV-encoded dUTPase-injected (G) NZM2410 mice exhibited a comparable degree of TLR2 expression, thus demonstrating that the glomerular up- regulation is EBV-dUTPase-mediated.

Since EBV-dUTPase is a strong activator of TLR2 [17,18,21] we next sought to study the potential involvement of TLR2 in EBV-dUTPase mediated enhancement of glomerulonephritis. While endogenous TLR2 expression was observed in the tubules of all treatment groups as expected; EBV-dUTPase injections significantly heightened the expression of TLR2 in the glomerulus when compared to control group (Figure 4A Figure 4B, Figure 4C and Figure 4D). Specificity of immunohistochemical staining was confirmed in salivary gland tissue sections present on the same slide, which also displayed positive TLR2 staining (Figure 4F and Figure 4G). Quantitative analysis of glomerular regions demonstrated significant differences in TLR2 expression between the EBV-dUTPase and PBS treatment groups (*p* = 0.0001; Figure 4E). Rabbit pre-immune serum and mouse control serum, used as negative controls, did not bind to renal glomeruli and tubules (data not shown). These findings are consistent with recent reports supporting a role for TLR2 in the formation of autoantibodies in SLE and suggest a role for EBV-dUTPase in the pathogenesis of LN. Furthermore, our data suggest that the dUTPase may contribute to the aberrant immune response observed in LN by inducing/maintaining a high expression of TLR2, which in turn drives/promotes the progression of the disease to a more severe phenotype.

### EBV-dUTPase stimulates IL-17 production in the kidneys of mice with LN

**Figure 5:**
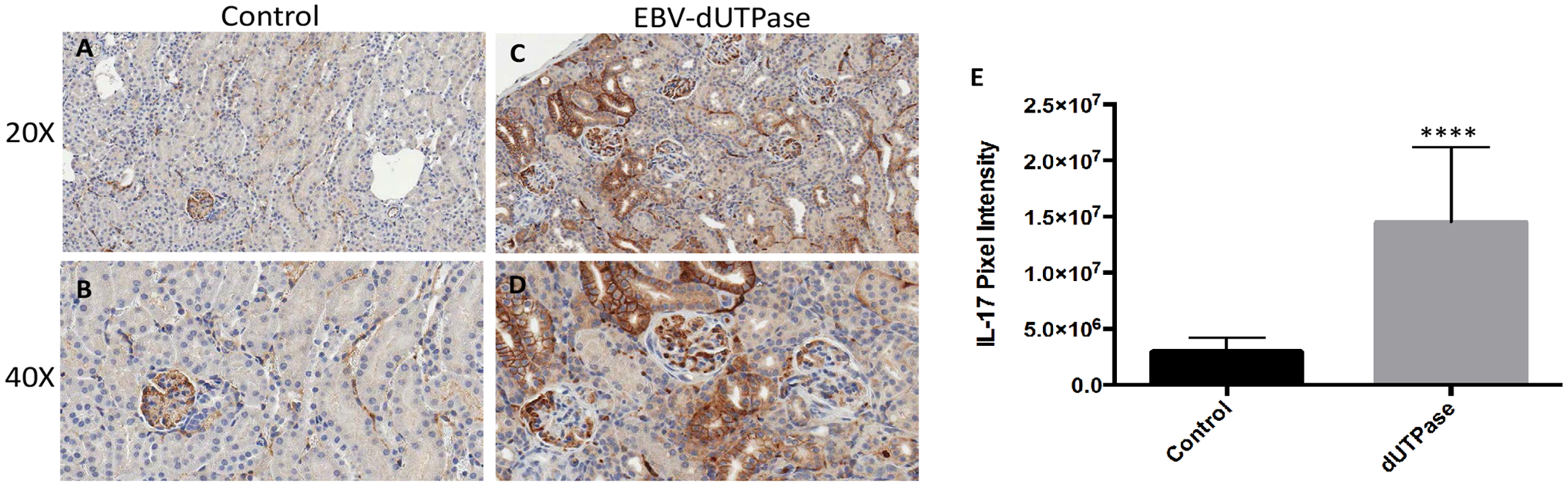
EBV-dUTPase protein is a strong inducer of IL-17 *in vivo*. Kidney sections from control (A,B) and EBV-dUTPase-injected (C,D) NZM2410/J mice were stained to demonstrate IL-17 protein expression (B,D). Hematoxylin counterstain. Original objective magnification: 20 × (A,B); 40 × (C,D). (E) Semi-quantitative analysis of kidney sections using the Aperio digital image analysis software demonstrated significant differences in the level of IL-17 protein expression between EBV-dUTPase-treated and PBS-injected mice. Values represent the mean pixel intensity of positive staining ± SD of n = 5 mice per treatment group; ****p < 0.0001 versus PBS control.

Activation of TLR2 by EBV-dUTPase results in the production of pro-inflammatory TH1/TH17 cytokines, which have been implicated in SLE pathogenesis. To determine whether the dUTPase from EBV contributes to the inflammatory responses observed in NZM2410/J mice by triggering the production of IL-17, we examined kidney sections from EBV-dUTPase or PBS injected mice for IL-17 protein by immunohistochemical analysis. Treatment of mice with EBV-dUTPase induced a strong and statistically significant increase of IL-17 in the glomerulus and tubules relative to control mice (Figure 5A, Figure 5B, Figure 5C and Figure 5D) (*p* < 0.0001; Figure 5E). Rabbit pre-immune serum, used as a negative control, did not detect IL-17 (data not shown).

### EBV-dUTPase is detected in plasma cells of infiltrates within kidneys of LN patients

**Figure 6:**
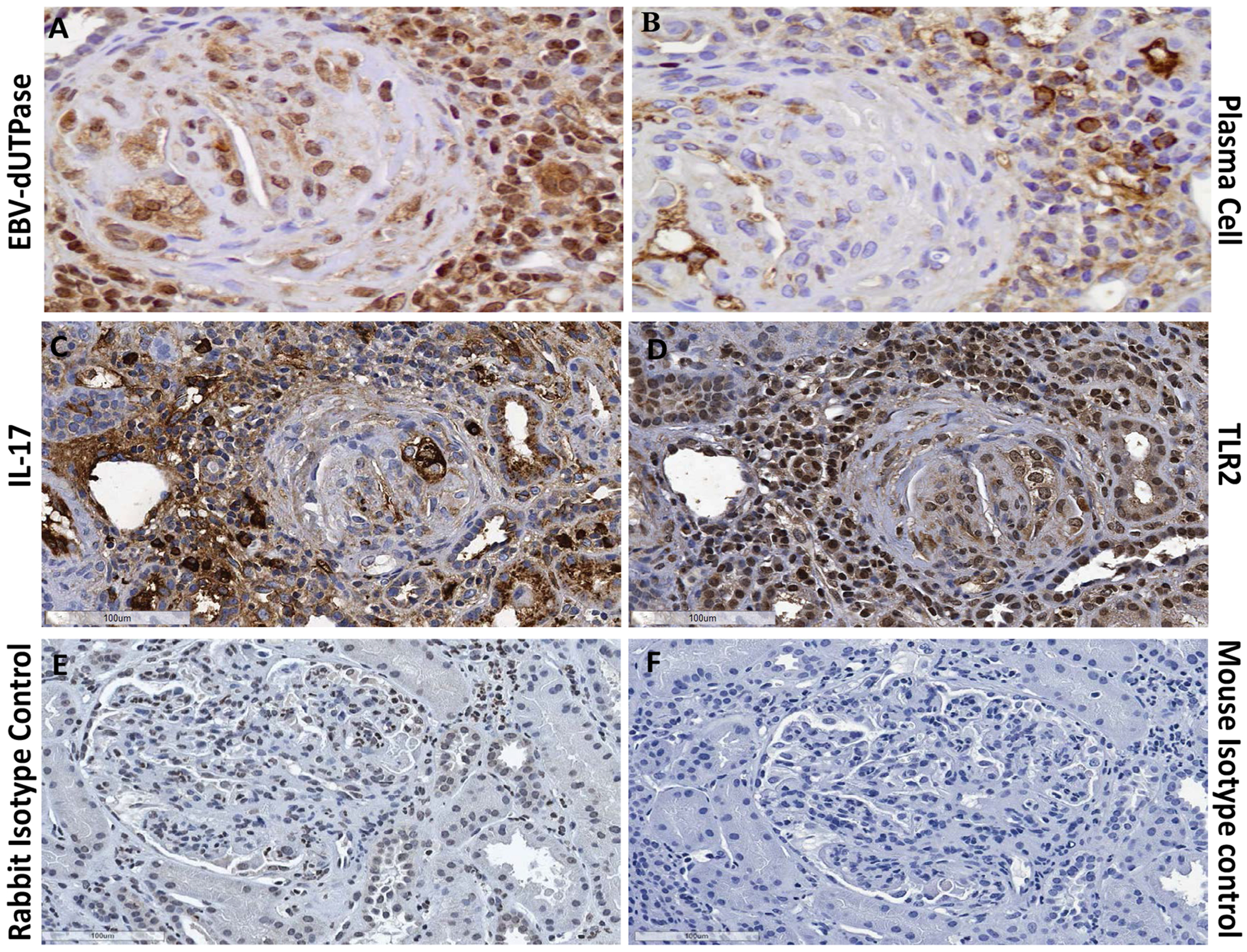
EBV-dUTPase protein is expressed in kidney biopsies of SLE patients with LN in infiltrates containing plasma cells. Kidney sections from patients with class III or IV LN were stained for the presence of EBV-dUTPase protein (A), plasma cells (B), TLR2 (C), or IL-17 (D). Rabbit pre-immune serum or mouse isotype control antibody (E, F) were used as negative controls. Hematoxylin counterstain images shown are representative of trends observed in all patients.

We next examined kidney biopsy tissue obtained from SLE patients (n = 6) with class III or IV LN who were undergoing flares. Immunohistochemical staining identified the presence of EBV-dUTPase protein in infiltrating lymphocytes and within the glomerulus of patients with LN (Figure 6A). Furthermore, the positive staining localized to areas that are enriched with plasma cells (Figure 6B), which is consistent with the known abortive-lytic/lytic replication pattern of EBV in these cells, and cells expressing TLR-2 and IL-17 (Figure 6C and Figure 6D, respectively). Rabbit pre-immune serum or mouse isotype control Ab, used as negative controls, did not detect expression of viral dUTPase, TLR2 or IL-17 (Figure 6E and Figure 6F, respectively). Similar results were observed in all six SLE patients’ kidney tissue examined. While healthy adults and lupus patients who are seronegative for the virus are extremely rare, it would be interesting to determine whether LN patients who are EBV seropositive in remission are expressing EBV-dUTPase.

## Discussion

EBV, which infects a significant percentage (> 90%) of the world’s population and establishes a life-long persistent infection, is implicated in the pathogenesis of human epithelial and hematopoietic malignancies and several autoimmune diseases including SLE, multiple sclerosis, and chronic fatigue syndrome. However, EBV’s role in autoimmunity remains poorly understood. The current study provides evidence supporting a role for EBV-dUTPase in the exacerbation of glomerulonephritis and renal pathology in a mouse model of LN, as indicated by the significant increase in macrophage infiltration, IgG complex formation and complement (C3) deposition in the kidneys of dUTPase-treated mice (Figure 2 and Figure 3). Immunohistochemical analysis of kidney biopsy tissue from LN patients (class III and IV) undergoing flares also demonstrated the expression of EBV-dUTPase in plasma cell aggregates near glomeruli (Figure 6). LN patients undergoing flares exhibit an enrichment of long-lived autoantibody producing memory plasma cells in the kidneys [26], which are believed to contribute to disease pathogenesis. Interestingly, the terminal differentiation of memory B-cells into plasma cells causes the reactivation of latent EBV resulting in the expression of the dUTPase [27,28]. More importantly, we have previously demonstrated that the dUTPase protein is released in exosomes from EBV-infected B cells (plasma cells) during abortive lytic replication of EBV and these dUTPase-containing exosomes induced the production of pro-inflammatory cytokines in DC and PBMCs by ligation of TLR2 [18].

There is increasing evidence from murine models [22,29–33] and human LN studies [4,32,33] that TLR2 and the pro-inflammatory cytokine IL-17 may play a role in the pathophysiology of LN. In support of this premise, recent studies have demonstrated that the TLR2/MyD88/miRNA155/Ets-1 pathway is required for the production of autoantibodies that form DNA-containing immune complexes [22] and that increased TLR2 expression promotes IL-17 production in SLE patients [33]. Furthermore, a recent study has shown that IL-17 serum levels are statistically increased in patients with SLE and exhibited a positive correlation with SLE Disease Activity Index (SLEDAI) scores [34]. In line with these findings, extensive studies by our group have demonstrated that the EBV-dUTPase activates TLR2 in human embryonic kidney cells in a dose-dependent manner [17,21], and that EBV-dUTPase-mediated activation of TLR2 on immune cells results in the production of pro-inflammatory TH1/TH17 cytokines [20,21], which have been implicated in the pathogenesis of SLE [29,30]. We sought to determine whether EBV-dUTPase-mediated enhancement of glomerulonephritis may involve TLR2 and the production of IL-17. The data presented in this study demonstrate that there is a statistically significant up-regulation of TLR2 and IL-17 expression in the tubulointerstitium and glomeruli of NZM2410/J mice injected with EBV-dUTPase. Positive staining for TLR2 and IL-17 was also observed in kidney biopsy tissue from LN patients (class III and IV) undergoing flares. These data are consistent with literature reports suggesting that activation of TLR2 is necessary toenhance glomerulus inflammation, the production of autoantibodies against dsDNA, hallmarks of LN, and IL-17.

We hypothesized that the reactivation of EBV in LN patients with active disease and in those undergoing flares [35] would lead to the expression of the *BLLF3* gene of EBV, which encodes for dUTPase. Subsequent dUTPase expression could be enhanced by the infection of renal tubular epithelial cells, which also results in abortive and productive lytic infection [36]. Our results suggest that EBV-dUTPase may contribute to the continuous immune activation observed in a subset of LN patients by signaling through the innate immune receptor TLR2, which could lead to increased autoantibody production through the TLR2/MyD88/miRNA155/Ets pathway and drive the differentiation of naïve CD4^+^ T-cells into TH17cells. Enhanced TH17 activation would then result in the increased expression of IL-17 and impairment of EBV-specific CD8^+^ T-cell function, leading to decreased immune surveillance of EBV and increased virus loads.

Collectively, our data indicates that it is unlikely that EBV acts as a trigger for SLE or LN, but rather reflects alterations in immune hemostasis/surveillance. However, our previous works, as well as, this study, provide important evidence supporting a role for EBV-dUTPase in the exacerbation of the immune pathology of LN. Furthermore, our results provide compelling evidence supporting the premise that the EBV-encoded dUTPase may be a novel target for the development of alternative therapeutic agents for LN.
